# Diagenetic nutrient supplies to the Proterozoic biosphere archived in divergent nitrogen isotopic ratios between kerogen and silicate minerals

**DOI:** 10.1111/gbi.12507

**Published:** 2022-06-24

**Authors:** Eva E. Stüeken, Anthony R. Prave

**Affiliations:** ^1^ School of Earth & Environmental Sciences University of St Andrews St Andrews UK

## Abstract

Nitrogen isotopes and abundances in sedimentary rocks have become an important tool for reconstructing biogeochemical cycles in ancient ecosystems. There are two archives of nitrogen in the rock record, namely kerogen‐bound amines and silicate‐bound ammonium, and it is well documented that the isotopic ratios of these two archives can be offset from one another. This offset has been observed to increase with metamorphic grade, suggesting that it may be related to the bonding environment in differing nitrogen host phases and associated equilibrium isotope fractionation. However, theoretical bounds for this effect have not been established, and it remains possible that some isotopic offsets predate metamorphism. In support of this hypothesis, we report an unexpectedly large isotopic offset of 4–5‰ in siltstones of very low metamorphic grade from the late Mesoproterozoic Diabaig Formation in NW Scotland (1.0 Ga). Carbon to nitrogen ratios of bulk rocks are 2–3 times lower than in other Mesoproterozoic sections. The rocks also contain early‐formed phosphate concretions and display wrinkled surfaces on bedding planes, indicative of fossilised microbial mats. Collectively, these data are most parsimoniously interpreted as evidence of diagenetic ammonium release from microbial mats into porewaters, followed by partial oxidation to nitrite or nitrate at the sediment–water interface. This process would render residual ammonium in clays isotopically heavy, while the resulting nitrite or nitrate would be relatively lighter and captured in new biomass, leading to the observed isotopic divergence. The same diagenetic degradation pathway likely also liberated phosphate that was trapped within concretions. Diagenetic release of nutrients is known to occur in modern settings, and our data suggest that nitrogen isotopes may be a way to track this local sedimentary nutrient source in past environments. Lastly, we speculate that diagenetic nutrient recycling within Proterozoic microbial mats may have created a favourable niche for eukaryotic organisms in shallow waters.

## 1 INTRODUCTION

Nitrogen isotopes (i.e. stable ^15^N/^14^N ratios, expressed in delta notation relative to modern air, δ^15^N [‰] = [(^15^N/^14^N)_sample_/(^15^N/^14^N)_air_ – 1] x 1000) have become an important tool for reconstructing the evolution of the biogeochemical nitrogen cycle over Earth's history (Ader et al., 2016; Stüeken et al., 2016). For example δ^15^N have been used to explore the growth and shrinkage of the marine nitrate reservoir in response to redox changes in the ocean (Ader et al., 2014; Johnson et al., 2017; Kipp et al., 2018; Koehler et al., 2019; Luo et al., 2016; Luo et al., 2018; Zerkle et al., 2017), the antiquity of biological metabolisms in the nitrogen cycle (Beaumont & Robert, 1999; Garvin et al., 2009; Godfrey & Falkowski, 2009; Stüeken et al., 2015), the expansion of ammonium in seawater (Higgins et al., 2012; Papineau et al., 2009; Yang et al., 2019) or the alkalinity of ancient lakes (Stüeken, Tino, et al., 2020; Talbot & Johannessen, 1992; Xia et al., 2022). Through this work, it has become apparent that nitrogen is stored in sedimentary rocks in two distinct archives: bound to organic matter or trapped within silicate minerals. Organic‐bound nitrogen is introduced into sediments through biomass burial, while the silicate‐bound fraction can be of either detrital origin, or it can form during diagenesis and metamorphism, as ammonium is released from biomass into porewaters and adsorbed to clay minerals (Müller, 1977; Rosenfeld, 1979; Schroeder & McLain, 1998). For example in modern anoxic porewaters, dissolved ammonium levels can reach mm concentrations, compared with roughly 30 μm of nitrate in open seawater (Boudreau & Canfield, 1988; Graca et al., 2006). Hence, the silicate‐bound N fraction can make up a large proportion of the total nitrogen contained within sedimentary samples (Stüeken et al., 2017). However, although silicate‐bound N forms ultimately from the diagenetic breakdown of biomass, some studies have found it to be isotopically distinct (Kipp et al., 2018; Stüeken et al., 2017). Systematic comparisons of the two nitrogen reservoirs within the same rocks revealed that the isotopic difference increases with metamorphic grade, where the kerogen‐bound fraction appears to become lighter while the silicate‐fraction becomes heavier (Kipp et al., 2018; Stüeken et al., 2017). The isotopic offset is on the order of 1–2‰ below greenschist facies and 2–4‰ at greenschist facies. This observation suggests that the bonding environment of nitrogen within the two host phases may change during progressive metamorphic alteration, leading to equilibrium isotopic fractionation.

However, it is known from modern environments that nitrogen species with differing redox states (nitrate, nitrite, organic amines and ammonium) and distinct isotopic compositions may transiently coexist within sediments and in the water column (Andrisoa et al., 2019; Morales et al., 2014; Prokopenko et al., 2006; Sun et al., 2021). If these species are trapped separately by clay minerals and living organisms, then it is conceivable that isotopic offsets between the silicate‐bound and organic‐bound N fractions are produced during the time of sediment deposition. Hence, such isotopic offsets may not necessarily be an artefact of metamorphic alteration alone but could in fact carry information about past environments. Here, we document isotopic offsets of up to 5‰ between the two nitrogen reservoirs in rocks of sub‐greenschist metamorphic grade from the late Mesoproterozoic Diabaig Formation of the Torridonian Supergroup in NW Scotland (1.0 Ga). These offsets are larger than expected for this low degree of metamorphic alteration. We therefore propose that they capture diagenetic diffusion of ammonium towards the sediment–water interface, where isotopic fractionations were induced by partial nitrification within oxygenic microbial mats. Paired with other observations from these rocks, the nitrogen data may thus provide a novel window into a potentially important mechanism of nutrient recycling on the Proterozoic Earth.

## 2 GEOLOGICAL SETTING

**FIGURE 1:**
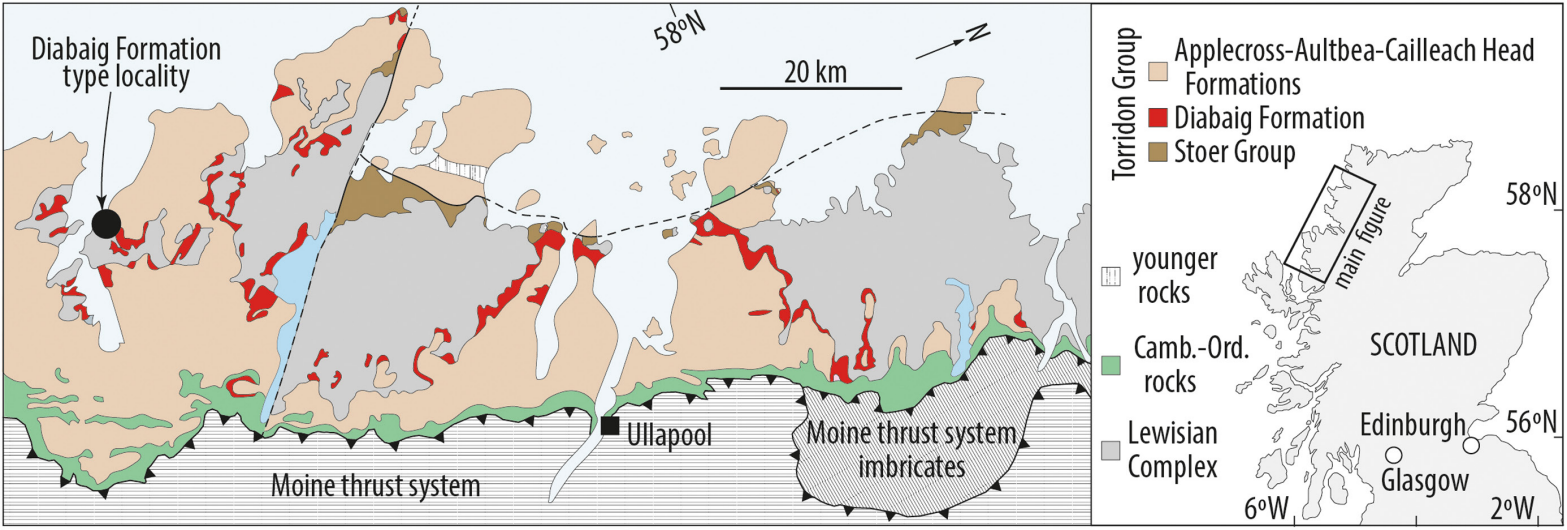
Simplified geological map and outcrop belt of the Diabaig formation, NW Scotland. Samples for this study were collected from the type locality

**FIGURE 2:**
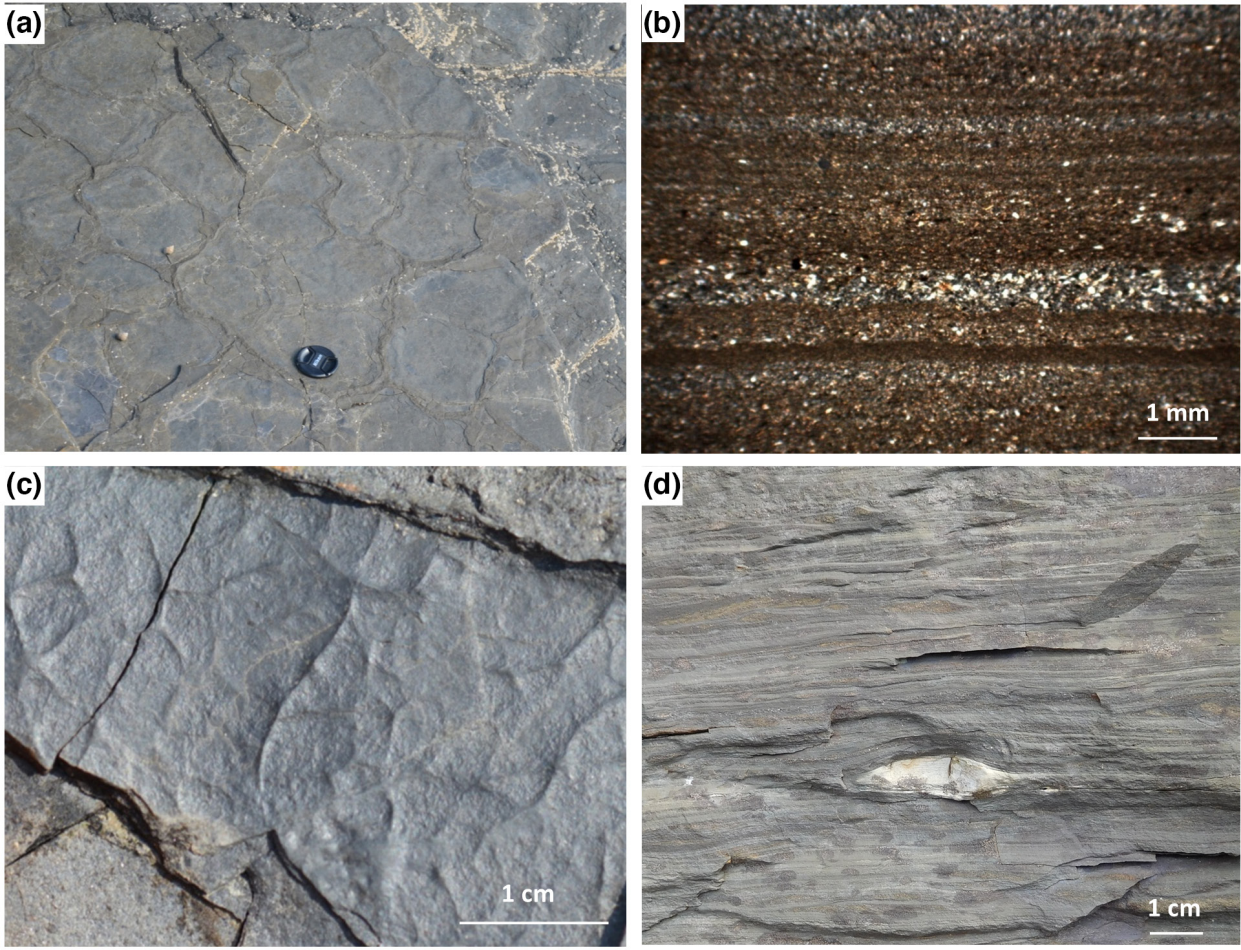
Images of the upper Diabaig formation. (a) Sand‐filled desiccation cracks formed in grey shale and siltstone (lens cap = 6 cm). (b) Photomicrograph of rhythmic interlaminated shale‐silt couplets; plane‐polarised light. (c) Wrinkled and microbial mat textures on a surface broken by syneresis cracks. (d) Light‐coloured phosphate nodule within grey shale and siltstone; note compaction of laminae around the nodule indicating that the timing of its formation was pre‐compactional

The late Mesoproterozoic Diabaig Formation is part of the Torridon Group in NW Scotland (1.0 Ga, Stewart, 2002; Figure 1). The Group is composed of siliciclastic sedimentary rocks that were deposited unconformably on Archean to Paleoproterozoic Lewisian gneiss. The Diabaig Formation is the lower‐most unit of the Group and in most places consists of a variably developed basal breccia that passes upwards into grey, interbedded fine sandstones and shales. Geochemical provenance analyses indicate that around 80% of the sediments in the Diabaig Formation were derived from the Lewisian basement (Rodd & Stewart, 1992; Young, 1999). At the type section (57.577°N−5.686°E), which was the focus of this study, a c. 20‐m‐thick basal breccia and pale red sandstone unit sits directly on the Lewisian basement; many of the sandstones are wave‐rippled with red mud drapes. The majority of the Formation is a c. 120 m thick siltstone‐shale unit that is grey in colour and characterised by abundant desiccation cracks, mm‐ to cm‐scale wave and current ripples and rare phosphate concretions (Figure 2). Cyanobacterial mats and eukaryotic acritarchs have been well documented from this part of the Formation (Callow & Brasier, 2011; Strother et al., 2011). The topmost c. 40 m of the Diabaig type section consists of dm‐thick trough cross‐bedded sandstones interbedded with grey shales. Collectively, these observations reveal a shallow‐water setting that was frequently exposed to the atmosphere and extensively covered with bacterial and algal biomass. Previous workers proposed that the Diabaig Formation was deposited in a lacustrine basin, based on the boron content of illite separates and the proximity to fluvial sandstones (Stewart, 2002; Stewart & Parker, 1979). We stress that the palaeoenvironmental setting of the Diabaig rocks, whether freshwater, brackish or marine, is not an issue because the diagenetic processes documented herein are not restricted by salinity.

## 3 METHODS

We collected 51 samples of grey siltstones and shales for analyses of carbon and nitrogen isotopes as well as major elemental abundances. Furthermore, we collected seven samples from the Lewisian basement, including three samples of quartz‐feldspar‐biotite gneiss and four samples of amphibolite, for nitrogen abundance measurements to assess potential contributions of detrital N in our sedimentary samples. Outer surfaces of the samples were trimmed with a water‐cooled rock saw and the interiors were hammered into sub‐cm sized chips. The chips were cleaned sequentially in methanol, 1 m HCl (both reagent grade) and 18 MΩ cm^−1^ DI water, left to dry overnight in a closed oven and then pulverised in an agate ball mill. The powder was stored in pre‐combusted glass vials (500°C overnight). For analyses of total organic carbon (TOC), carbon isotopes and bulk nitrogen, c. 0.5 g of each sample, was treated with 10 ml of 2 m HCl at 60°C in two iterations to remove carbonate. The decarbonated powders were washed three times with DI water and left to dry at 60°C. For a subset of samples, we also extracted kerogen to be able to analyse kerogen‐bound nitrogen separately (Stüeken et al., 2017). Around 3–4 g of untreated rock powder were weighed into a 250 ml Teflon bottle and treated with mixture of 50 ml of concentrated HF and 50 ml of DI water at 50°C in a shaking water bath. After 1 day, the acid was decanted and replaced by 100 ml of dissolved BF_3_, which was prepared by dissolving 31 g of boric acid in 50 ml of DI water and 50 ml of HF. BF_3_ attacks secondary fluoride precipitates (Robl & Davis, 1993). After one more day, the BF_3_ solution was decanted, and the residue was washed three times with DI water. Residual water was removed with a freeze drier.

For isotopic analyses, decarbonated rock powders or kerogen extracts were weighed into tin capsules and flash‐combusted in an elemental analyzer (EA IsoLink), coupled via a ConFlo IV to a MAT253 isotope ratio mass spectrometer (Thermo Fisher Scientific). Isotopic ratios were calibrated with the international reference materials USGS‐40 and USGS‐41. Signal intensities of the standards were used to calibrate for elemental abundances. The results are expressed in standard delta notation (δ = [R_sample_/R_standard_ − 1] x 1000), where R = ^13^C/^12^C or ^15^N/^14^N. Reference standards are VPDP for carbon and atmospheric air for nitrogen. Average reproducibility for our samples was 0.3‰ for δ^13^C_org_, 0.3‰ for δ^15^N_bulk_ and 0.1‰ for δ^15^N_ker_. We have previously validated this method with international shale standards (SGR‐1 and SDo‐1) and found good agreement with published values (Dennen et al., 2006; Stüeken, de Castro, et al., 2020). The most recent analyses of non‐decarbonated aliquots of these standards (SGR‐1: δ^15^N = 17.94 ± 0.04 ‰, TN = 0.83 ± 0.001 wt. %, *n* = 3; SDo‐1: δ^15^N = −0.60 ± 0.38‰, TN = 0.34 ± 0.002 wt. %, *n* = 3) also agree well (expected SGR‐1: δ^15^N = 17.4 ± 0.4‰, TN = 0.81 ± 0.02 wt. %,; SDo‐1: δ^15^N = −0.8 ± 0.3‰, TN = 0.36 ± 0.01 wt. %; Dennen et al., 2006), indicating that our analytical protocol is reliable. Kerogen was also analysed for hydrogen contents to calculate organic H/C ratios as a proxy for metamorphic grade. These analyses were done with the elemental analyzer in stand‐alone mode using the thermal conductivity detector. Sulphanilamide was used for calibration. Replicate analyses of standards treated as unknowns showed a precision of better than ±2%.

We also analysed inorganic carbon and oxygen isotopes in two samples of a carbonate‐cemented sandstone. Untreated powder aliquots were weighed into acid‐washed Exetainer vials, capped with a rubber septum and purged with helium for 10 min. Ten drops of concentrated phosphoric acid were injected with a syringe, and the evolved CO_2_ gas was sampled from the headspace of the vial with a Gasbench II, coupled to a Delta Plus XP isotope ratio mass spectrometer (Thermo Fisher Scientific). NBS‐19 and NBS‐18 were used for calibration to the VPDB scale. The reproducibility was 0.1‰ for δ^13^C_carb_ and 0.2‰ for δ^18^O_carb_. A subset of samples was sent to ALS for bulk rock analyses of metal abundances. Here, the samples were dissolved in HNO_3_, HF and HClO_4_, treated with HCl to remove fluorides and analysed by inductively coupled plasma mass spectrometry and optical emission spectroscopy. Relative errors (standard deviation/mean) were 5% or better. OREAS‐45d and OREAS‐905 were used for quality control and found to agree well with accepted values.

## 4 RESULTS

**FIGURE 3:**
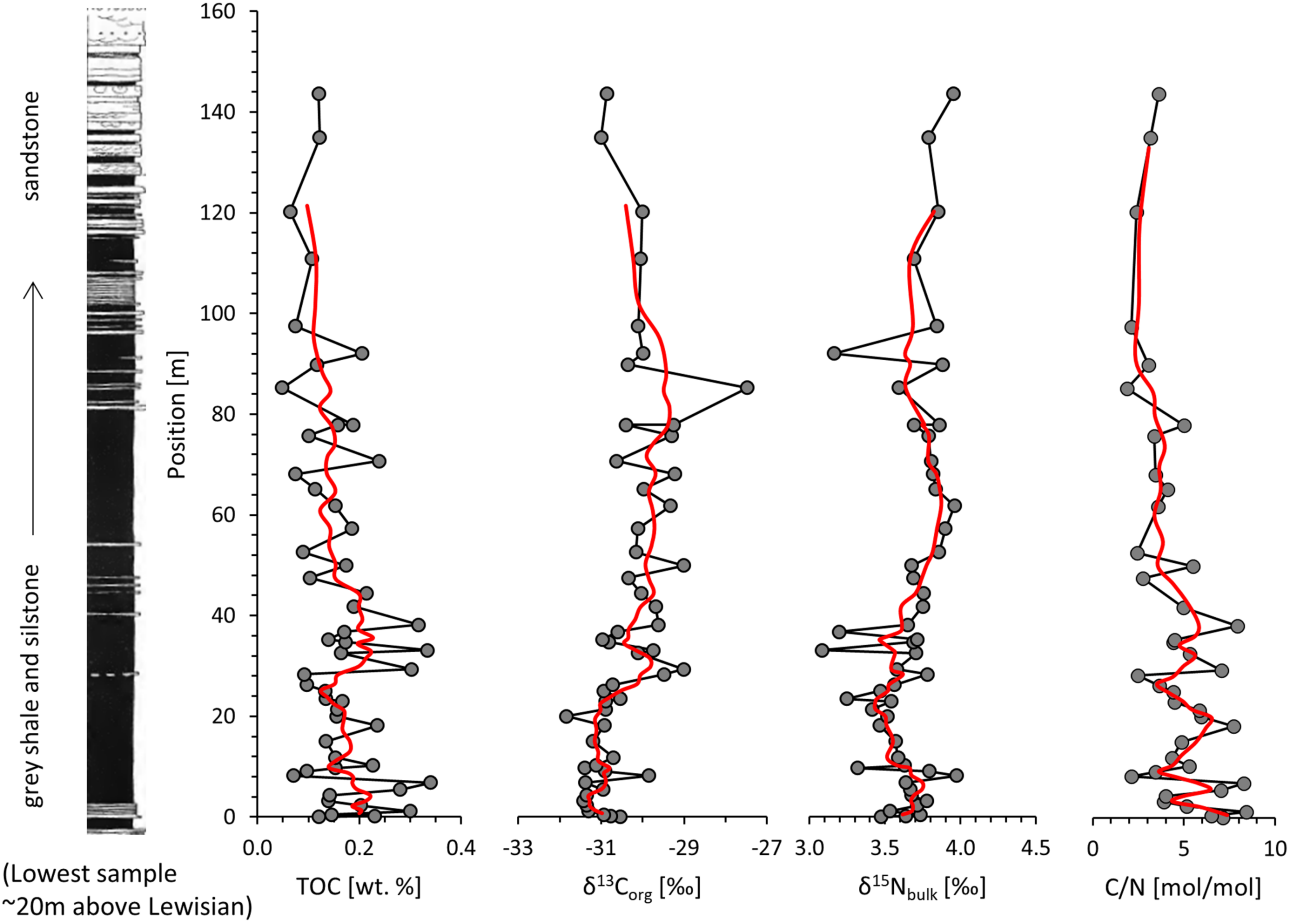
Diabaig formation type locality (after Stewart, 2002) and accompanying analyses. Red line = 3‐point running mean. Lithology: Black = interlaminated siltstone and shale; white = sandstone

**FIGURE 4:**
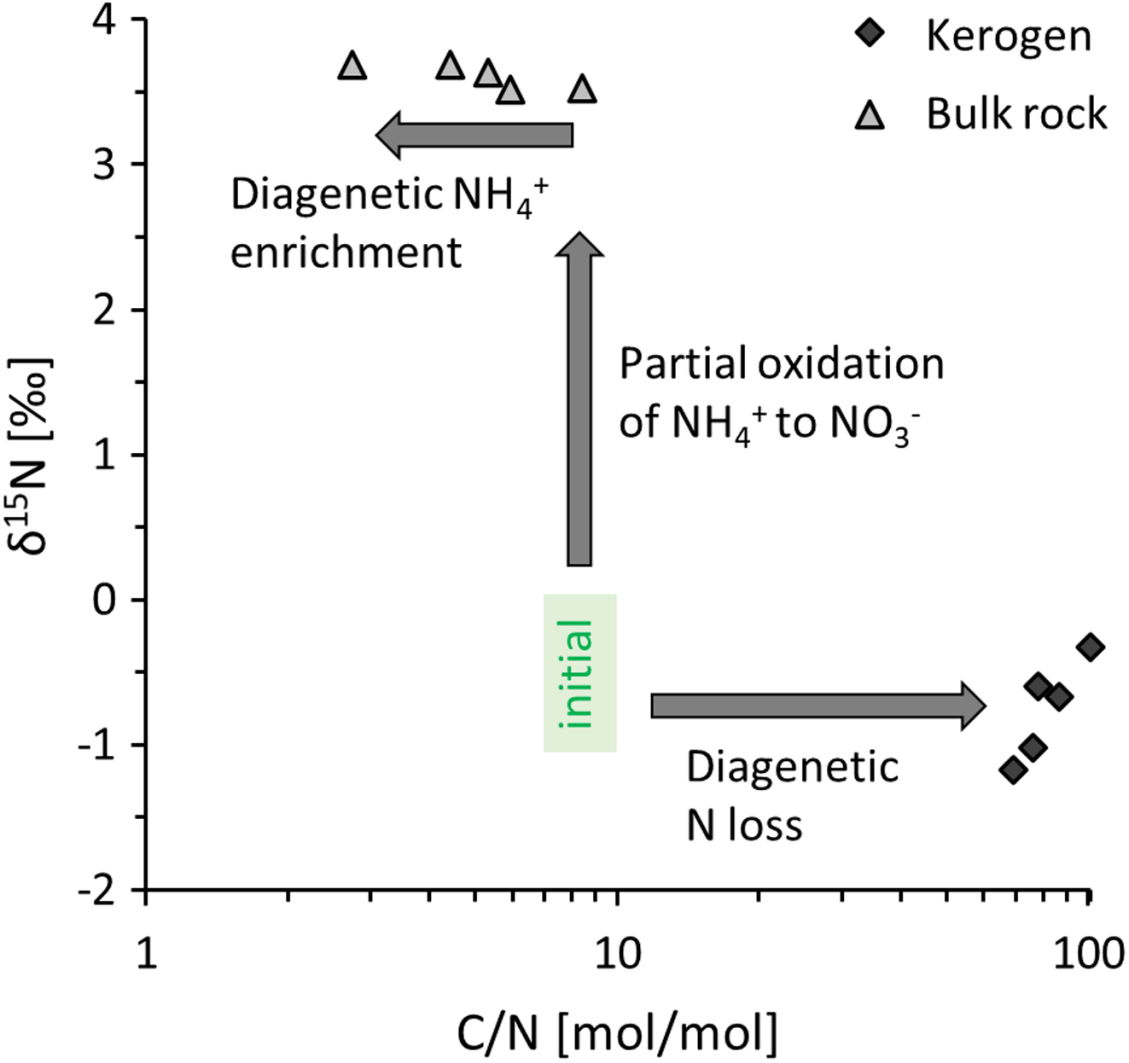
Nitrogen isotopes versus organic carbon to nitrogen ratios for bulk rock samples (triangles) and kerogen isolates (diamonds). The initial value of bulk nitrogen in the system is assumed to fall within the isotopic range of diazotrophs (−1‰ to 0‰) with a C/N ratio (7–10) equivalent to that of modern bacteria (Godfrey & Glass, 2011; Sigman et al., 2001)

Total organic carbon (TOC) concentrations in the Diabaig Formation fall between 0.1 and 0.2 wt.% (Figure 3, Table 1), and total nitrogen concentrations show a mean of 393 ± 67 μg/g. Molar organic carbon to nitrogen ratios (C/N) of bulk rocks fall between 4 and 6 and are thus low compared with a Mesoproterozoic median of 15 (Stüeken et al., 2016). Organic carbon isotopes (δ^13^C_org_) range from −31.5‰ to −29‰, and the two measurements of carbonate‐cemented sandstones gave δ^13^C_carb_ values of −3‰ and −5‰. Bulk nitrogen isotopes (δ^15^N_bulk_) show little variability across the formation with a range from +3.4‰ to +4.0‰. Kerogen extracts from five samples show δ^15^N_ker_ values with an average of −0.8 ± 0.3‰ (Figure 4, Table 2), which is about 4–5‰ lower than the corresponding bulk rock analyses. Mass balance calculations indicate that 90%–96% of the total nitrogen is silicate‐bound. Molar organic H/C ratios range from 0.40 to 0.43, which corresponds to a prehnite‐pumpellyite facies metamorphic grade (Hayes et al., 1983). At such a low metamorphic grade, the observed offset between kerogen and bulk nitrogen isotope ratios is thus larger than the expected 1–2‰ that is observed elsewhere (Stüeken et al., 2017). Bulk Fe/Al ratios are fairly invariable with a mean of 0.57 ± 0.03 g/g (Table 3), which falls within the range of average soils (0.47 ± 0.15 g/g, Cole et al., 2017) and below the empirically defined threshold of 0.66 g/g above which depositional environments may be identified as anoxic (Raiswell et al., 2019). These rocks are thus neither enriched nor depleted in iron. Total phosphorus concentrations range from 0.033 wt. % to 0.237 wt. %. Samples without visible phosphate concretions show an average concentration of 0.062 ± 0.020 wt. %, which is at the upper end of average sedimentary phosphorus concentrations through the Precambrian (0.04 + 0.04/−0.02 wt.%, Reinhard et al., 2017). The seven samples from the Lewisian basement (Table 4) showed very low total N abundances of less than 10 μg/g. These levels were too low for isotopic analyses.

**TABLE 1:** Bulk rock organic carbon and nitrogen data from the Diabaig type locality

Position [m]	TOC [wt. %]	δ^13^C [‰]	C/N [mol/mol]	TN [μg/g]	δ^15^N [‰]
0.00	0.23	−30.77	7.1	378	3.64
0.10	0.12	−30.54	4.2	331	3.48
0.46	0.15	−30.93	6.5	261	3.73
1.14	0.30	−31.30	8.4	414	3.53
2.28	0.20	−31.35	5.1	459	3.71
3.19	0.14	−31.43	3.9	417	3.78
4.32	0.14	−31.36	4.0	414	3.67
5.46	0.28	−30.95	7.0	465	3.67
6.83	0.34	−31.38	8.3	479	3.64
8.19	0.07	−29.85	2.1	392	3.97
9.10	0.10	−30.91	3.4	331	3.79
9.77	0.15	−31.40	5.1	350	3.32
10.24	0.23	−31.12	5.3	499	3.63
11.83	0.15	−30.71	4.4	410	3.59
15.02	0.13	−31.19	4.9	322	3.57
18.21	0.24	−30.92	7.7	357	3.47
20.03	0.16	−31.83	5.9	306	3.52
21.39	0.16	−30.90	5.9	311	3.41
22.99	0.17	−30.89	4.5	433	3.54
23.45	0.13	−30.53	3.7	418	3.25
25.04	0.13	−30.93	4.4	350	3.47
26.28	0.10	−30.72	3.6	310	3.56
28.30	0.09	−29.48	2.5	435	3.78
29.32	0.30	−29.02	7.0	500	3.58
32.55	0.16	−30.11	5.3	360	3.71
33.13	0.33	−29.74	8.6	456	3.08
34.77	0.17	−30.82	4.4	454	3.69
35.29	0.14	−30.97	4.5	364	3.71
36.76	0.17	−30.59	3.3	604	3.20
38.14	0.32	−29.62	7.9	465	3.65
41.80	0.19	−29.69	5.0	442	3.75
44.44	0.21	−30.03	4.9	515	3.76
47.50	0.10	−30.34	2.7	440	3.69
49.95	0.17	−29.01	5.5	369	3.68
52.61	0.09	−30.16	2.4	429	3.86
57.24	0.19	−30.11	4.4	494	3.90
61.81	0.15	−29.33	3.6	495	3.96
65.15	0.11	−29.98	4.1	323	3.84
68.13	0.07	−29.22	3.5	253	3.82
70.72	0.24	−30.63	6.6	422	3.81
75.73	0.10	−29.29	3.4	347	3.79
77.89	0.16	−30.41	3.8	482	3.69
77.91	0.19	−29.25	5.0	437	3.86
85.24	0.05	−27.47	1.9	302	3.59
89.85	0.12	−30.35	3.1	446	3.88
92.08	0.21	−29.99	5.0	477	3.16
97.45	0.07	−30.11	2.1	410	3.84
110.83	0.11	−30.05	3.8	329	3.69
120.25	0.06	−30.01	2.4	311	3.85
134.91	0.12	−31.00	3.2	450	3.79
143.60	0.12	−30.86	3.6	387	3.95

**TABLE 2:** Kerogen data from selected samples. Bulk rock data are the same as in Table 1

Position [m]	TOC_bulk_ [wt. %]	TN_bulk_ [μg/g]	δ^15^N_bulk_ [‰]	TOC_ker_ [wt. %]	TN_ker_ [μg/g]	δ^15^N_ker_ [‰]	H/C_ker_ [mol/mol]
1.14	0.30	414	3.53	63.58	8567	−0.67	0.40
10.24	0.23	499	3.63	64.05	9544	−0.60	0.40
20.03	0.16	306	3.52	61.14	9364	−1.02	0.43
34.77	0.17	454	3.69	59.99	6940	−0.33	0.40
47.50	0.10	440	3.69	45.51	7643	−1.17	0.40

**TABLE 3:** Major elemental abundances in selected samples

Position [m]	Al [wt. %]	Ca [wt. %]	Fe [wt. %]	K [wt. %]	Na [wt. %]	P [wt. %]	S [wt. %]
0.10	7.88	0.7	4.76	2.56	1.425	0.033	0.02
23.45	8.42	1.28	4.45	2.49	1.55	0.075	0.02
36.76	8.93	0.78	5.11	2.87	1.335	0.071	0.02
57.24	8.74	1.03	4.85	2.82	1.375	0.237	0.01
77.89	8.73	1.43	4.72	3.31	1.355	0.44	0.01
110.83	7.96	0.75	4.86	2.99	1.565	0.07	0.01

**TABLE 4:** Nitrogen abundances in Lewisian basement rocks collected from outcrops in the vicinity of the Diabaig type section

Sample ID	Lithology	TN
		[μg/g]
140912‐22A	Gneiss	<1
140912‐22C	Amphibolite	5.7
140913–4	Amphibolite	8.9
140913‐5A	Gneiss	9.3
140913‐5B	Gneiss	6.3
140914‐24A	Amphibolite	3.2
140914‐24B	Amphibolite	4.9

## 5 DISCUSSION

The Diabaig Formation stands out from other mid‐Proterozoic successions for at least five reasons: (1) it shows abundant evidence of exposure to the atmosphere as evident by ubiquitous desiccation cracks (Figure 2a), and yet the rocks themselves are unoxidised; (2) it is known for diverse assemblages of cyanobacterial and eukaryotic microfossils (Callow & Brasier, 2011; Strother et al., 2011); (3) it is relatively enriched in phosphate with notable concretions, whereas most sedimentary rocks in the Proterozoic are P‐depleted (Reinhard et al., 2017); (4) organic carbon to nitrogen ratios are relatively low compared with the Mesoproterozoic median, and the isotopic offset between kerogen and silicate‐bound nitrogen is larger than expected for this low metamorphic grade (Stüeken et al., 2016); and (5) it is bounded above and below by successions of red beds many 100 s of metres thick. We propose that these features identify the Diabaig rocks as providing a rare window into a combination of early diagenetic processes that released nutrients back into shallow‐water habitats as manifested in the relatively large δ^15^N offset between silicate‐ and organic‐bound nitrogen.

### 5.1 Diagenetic iron reduction

Most of the Torridon Group is characterised by haematite‐coated red sandstones and mudrocks, where the haematite was likely produced during oxidative weathering of the Lewisian basement (Rodd & Stewart, 1992). One can therefore assume that oxidised iron was similarly delivered into the Diabaig depositional system. The absence of this phase from the grey siltstones today suggests that it was reduced to ferrous iron, consistent with the presence of organic matter in these rocks. This organic matter likely occurred in the form of microbial mats, as evident by the abundance of wrinkled surfaces (Figure 2c), and these would have acted as a reductant during diagenesis. A similar interpretation has been put forward by Wacey et al. (2014), who proposed anoxia within sediments as a means of preserving microfossils. Indeed, δ^13^C_org_ values down to −31.5‰, that is slightly below the expected range of −28‰ to −30‰ for primary producers (Hayes et al., 1999), may reflect some degree of anaerobic secondary productivity. Similarly, the relatively low δ^13^C_carb_ values of −3‰ to −5‰ in the carbonate‐cemented sandstone are compatible with diagenetic contributions of dissolved inorganic carbon, such as diagenetic oxidation of organic carbon coupled to iron oxide reduction. For comparison, open‐marine carbonates from other basins of this age display δ^13^C_carb_ values that fall mostly within the range of 0‰ to +2‰ with rare excursions down to −2‰ (Kah et al., 2012), meaning that the carbonate cements in the Diabaig sandstones have received at least some input of isotopically light dissolved inorganic carbon, likely during diagenesis.

However, near‐crustal Fe/Al ratios (0.57 ± 0.03 g/g, Table 4) indicate that the resulting ferrous iron was not exported from the sediments. Pyrite is rarely observed in these rocks, suggesting that pyrite formation was not a major iron sink. Instead, we propose that ferrous iron got trapped in diagenetic chlorite and phosphate minerals both of which are present in the Diabaig rocks (Rodd & Stewart, 1992; Wacey et al., 2014). Phosphate occurs as interstitial cements and locally as cm‐sized lenticular francolite concretions that formed prior to sediment compaction and are thus early‐diagenetic. They are also enriched in carbonate (Rodd & Stewart, 1992; Wacey et al., 2014). The general scarcity of P in other Precambrian sedimentary rocks is thought to reflect efficient scavenging of dissolved phosphate into ferrous minerals and carbonates (Reinhard et al., 2017). The Diabaig rocks may thus be preserving direct evidence of this process, likely coupled with diagenetic iron reduction.

### 5.2 Diagenetic ammonium oxidation

While iron reduction coupled to biomass degradation oxidises organic carbon to CO_2_, iron‐driven oxidation of ammonium to nitrogen oxides, such as nitrite or nitrate, is thermodynamically unfavourable (ΔG_r_ > 0) above pH 6 (Stüeken et al., 2016). Ammonium would thus have accumulated in pore waters during biomass degradation (Equation 1), as observed in modern anoxic mud where it can reach millimolar concentrations (Boudreau & Canfield, 1988).

(1) $${\left({\mathrm{CH}}_{2}O\right)}_{106}{\left({\mathrm{NH}}_{3}\right)}_{16}\left(H_{3}{\mathrm{PO}}_{4}\right)\to 53{\mathrm{CO}}_{2}+53{\mathrm{CH}}_{4}+16{\mathrm{NH}}_{3}+H_{3}{\mathrm{PO}}_{4}$$

From pore waters, ammonium can become incorporated into phyllosilicate minerals in substitution for potassium (Schroeder & McLain, 1998). Illite, a major diagenetic ammonium sink, has been documented from the Diabaig shales (Rodd & Stewart, 1992). Anaerobic ammonium production, accumulation in pore waters and incorporation into clays could thus explain the relatively low C/N ratios in these rocks. Alternatively, low C/N ratios could result from a large contribution of detrital N contained in allochthonous minerals, in particular potassic micas and feldspars. In the case of the Diabaig Formation, the majority of detritus is thought to be derived from the Lewisian basement (Rodd & Stewart, 1992; Young, 1999). However, the Lewisian samples that we analysed contained only very low levels of nitrogen (<10 μg/g), which would imply that less than 3% of the average N content in the Diabaig samples is of detrital origin. Hence, a detrital explanation for the low C/N ratios is unlikely in this setting. It is also unlikely that this diagenetic ammonium enrichment was caused by unusually high abundances of clay minerals (or detrital micas), because the average Al content of the Diabaig samples (8.4 ± 0.4 wt. %) overlaps with that of upper continental crust (8.2 ± 0.4 wt.%, Rudnick & Gao, 2014). Hence the most parsimonious explanation for these low C/N ratios is diagenesis, where ammonium from degrading biomass was trapped underneath microbial mats and therefore able to build up to a relatively large reservoir within sedimentary pore fluids.

**FIGURE 5:**
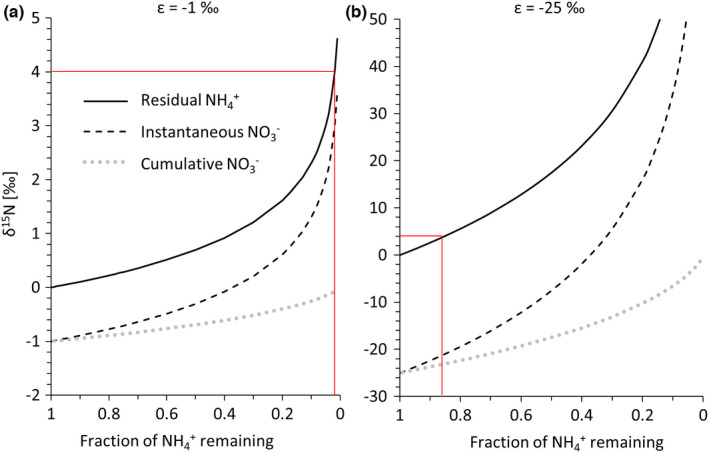
Rayleigh fractionation model of partial ammonium oxidation to nitrate with a net fractionation factor of −1‰ (a) and −25‰ (b). The residual ammonium becomes isotopically enriched during this process. The actual fractionation factor likely fell between these two endmembers

Diagenetic ammonium release and adsorption to clay minerals can thus explain the N abundance data; however, this process does not impart a significant isotopic fractionation (Koehler et al., 2019) and can therefore not explain the observed isotopic offset of over 4‰ between δ^15^N_silicate_ and δ^15^N_ker_. Such large offsets are observed at greenschist facies metamorphism or in rocks that have been permeated by hydrothermal fluids (Godfrey et al., 2013; Kipp et al., 2018; Stüeken et al., 2017), neither of which applies to the Diabaig Formation. We propose that this offset was instead caused by biological metabolisms acting on the diagenetic ammonium pool. One such process that could plausibly explain the data is partial ammonium oxidation to nitrate (nitrification) near the sediment–water interface. This reaction imparts a fractionation of −1‰ to −25‰, where the residual ammonium becomes isotopically heavier (Casciotti, 2009). Nitrification requires O_2_, and nanomolar oxygen levels are sufficient to drive the reaction to completion (Lipschultz et al., 1990). The isotopic fractionation associated with nitrification is therefore usually not expressed. In the case of the Diabaig setting, O_2_ would have been actively produced during the day by cyanobacterial mats, relics of which are preserved on bedding planes (Callow & Brasier, 2011). Ammonium diffusing from anoxic pore waters up to this interface could have undergone nitrification, as documented from modern microbial mats (Fan et al., 2015). However, in the absence of bioturbation ammonium concentrations in pore waters would have been diffusion‐limited and may have built up to high enough concentrations such that nitrification did not go to completion. If only a small fraction of ammonium was oxidised near the sediment–water interface, this could explain the relatively high δ^15^N_silicate_ values of the residual (now clay‐bound) ammonium. For a fractionation factor of −1‰, over 98% of diagenetic ammonium would need to undergo oxidation to elevate the isotopic composition of the residual ammonium by 4‰ (Figure 5a), but for a fractionation factor of −25‰, only about 15% of the ammonium pool needed to be oxidised (Figure 5b). A fractionation factor in between these two endmembers would generate a significant source of nitrate to overlying waters while retaining a large proportion of ammonium within sediments, ultimately leading to strong enrichments in clays. The isotopically light nitrate that escaped from the sediments could have been either transported during flood events across the sediment interface or assimilated by algae in situ. The kerogen value thus likely represents a mixture of primary N_2_‐fixers and minor contributions of nitrate assimilators.

We can narrow down the amount of lost N, using the H/C ratios of the kerogen with a mean of 0.4. Buick et al. (1998) calculated that organic matter with such an elemental composition would originally have contained approximately six times more carbon. This carbon was lost during low‐grade metamorphism. If we thus multiply our measured TOC values by a factor of 6, the new average C/N ratio of the bulk rocks would be 32, that is 3–4 times higher than the expected Redfield ratio of 7–10 (Godfrey & Glass, 2011). In other words, these bulk rocks have retained 25%–33% of their original N inventory while the remainder was lost. Part of this loss would have occurred during low‐grade metamorphic heating, but part of it may have occurred during diagenetic nitrification.

An alternative diagenetic process to explain these data are partial assimilation of ammonium into biomass, which is associated with a fractionation of −27‰ (Hoch et al., 1992). Like nitrification, this process renders the residual ammonium pool isotopically enriched in ^15^N. We cannot rule out that this pathway existed; however, unlike the partial nitrification mechanism described above, it would imply that all ammonium was retained within sediments, and we would thus expect to see more isotopically depleted kerogen reflective of partial ammonium uptake. In contrast, the partial nitrification scenario implies leakage of isotopically light nitrate into the overlying water column, from where it may have been exported. This way, the kerogen data that fall around −1 ‰ likely record primarily the initial N_2_‐fixation process with minor contributions of assimilated nitrate.

### 5.3 Implications

**FIGURE 6:**
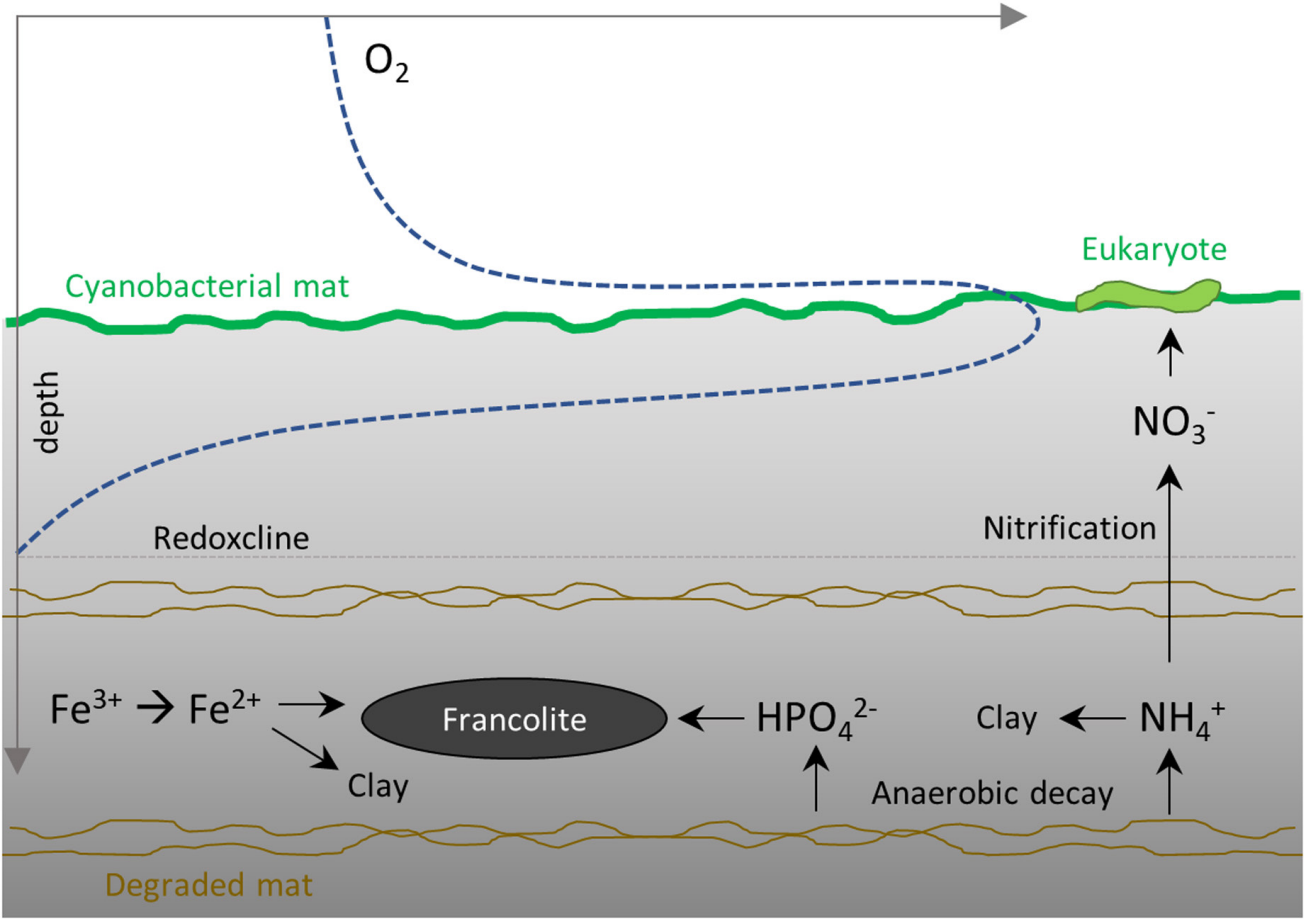
Schematic diagram of diagenetic processes. Ammonium would have been generated during anoxic biomass degradation within sedimentary pore waters. The absence of ferric iron in the Diabaig formation, compared with the surrounding red beds, supports this model of anaerobic diagenetic biomass degradation. If this ammonium diffused upwards towards the sediment–water interface, where microbial mats were actively producing O_2_, partial nitrification could have occurred, inducing the observed isotopic offset between silicates and kerogen. Nitrate escaping into the water column could have locally benthic eukaryotic algae. Phosphate released during the same diagenetic process was captured in francolite nodules

Our interpretation would imply that paired nitrogen isotope ratios of kerogen and silicate minerals in weakly metamorphosed sedimentary rocks can provide insights into diagenetic nitrogen recycling. On the modern Earth, the release and oxidation of ammonium from sediments is well documented (Graca et al., 2006; Grandel et al., 2000; Risgaard‐Petersen et al., 2004), and our approach may open the possibility of documenting such processes in deep time. During the Precambrian, diagenetic nutrient recycling may have been particularly important, because water columns were largely anoxic (Lyons et al., 2014) thereby prohibiting aerobic recycling of nutrients prior to settling on the seafloor (Kipp & Stüeken, 2017). The resulting nutrient scarcity may potentially have limited the proliferation of early life. Diagenetic processes that were able to provide nutrients locally would have created important ecological niches. Notably, findings of eukaryotic acritarchs in the mid‐Proterozoic are typically restricted to shallow‐water settings (Javaux, 2007), which are thought to have been relatively more oxygenated and nutrient‐rich compared with deeper waters below the photic zone (Koehler et al., 2017; Poulton & Canfield, 2011; Reinhard et al., 2016; Stüeken, 2013). We speculate that local upward diffusion and oxidation of nutrients from microbial mats, similar to the Diabaig Formation, may have elevated the habitability of such settings (Figure 6). Nitrate is a key source of nitrogen for eukaryotic algae because they are unable to perform N_2_ fixation and are easily outcompeted by bacteria for ammonium (Bouman et al., 2011; Glass et al., 2009; Karl et al., 2001). Hence the in situ production of nitrate at the sediment–water interface in shallow‐water microbial mats was perhaps an important nitrogen source for eukaryotic organisms at that time.

## 6 CONCLUSIONS

We conclude the following sequence of events for the deposition and diagenetic history of the Diabaig Formation that ultimately left a characteristic nitrogen isotope signature within those rocks:

1. Detrital material composed of partially oxidised siliciclastic material was eroded from the surrounding Lewisian basement and deposited in the basin. The red beds preserved in the lower part of the Diabaig Formation and those that typify the overlying Torridon Group retain this oxic detrital debris.
2. Microbial mats developed on sediment tops as evidenced by abundant wrinkled surfaces and cyanobacterial fossils (Callow & Brasier, 2011).
3. Anoxic conditions developed underneath these mats upon burial, inducing the reduction in ferric to ferrous iron. This diagenetic reduction reaction was coupled to anaerobic degradation of organic matter (C_org_ oxidation to CO_2_), releasing phosphate and ammonium into pore waters and creating isotopically depleted carbonates.
4. At least some of the phosphate was trapped in diagenetic iron phosphate minerals, which are visible as concretions and cements (Figure 2d).
5. Ammonium was partially oxidised to nitrate at the sediment–water interface within oxygenic microbial mats, leading to an isotopic enrichment in the residual ammonium in pore waters. This isotopically heavy ammonium residue became incorporated into potassic clay minerals while the isotopically lighter nitrite and nitrate were made available to organisms living near the sediment–water interface. As organic carbon was partially lost by oxidation to CO_2_, ammonium retention in clays lowered the total C/N ratio of sediments.

Diagenetic degradation of diazotrophic cyanobacterial mats, followed by partial oxidation to nitrate at the sediment–water interface, would thus have created a direct source of nitrate to benthic organisms. Microbial mats within the photic zone may therefore have represented important habitats for the development and diversification of eukaryotic algae, which today rely on a supply of nitrate. Lastly, our results provide a new interpretive framework for nitrogen isotopic offsets between silicate‐ and organic‐bound nitrogen in sedimentary rocks.

## CONFLICT OF INTEREST

We have no conflicts of interest to declare.

7

## Data Availability

The data that support the findings of this study are available in Tables 1, 2, 3, 4 of this article.
